# The Role of Radiofrequency Ablation in the Treatment of Trigeminal Neuralgia: A Narrative Review

**DOI:** 10.7759/cureus.36193

**Published:** 2023-03-15

**Authors:** Ebram Eskandar, Harendra Kumar, Aishwarya Boini, Felipe Velasquez Botero, Ghalib Nashaat El Hunjul, Maria A Nieto Salazar, Jonathan Quinonez, Bao Dinh, Joseph E Mouhanna

**Affiliations:** 1 Medical School, Philadelphia College of Osteopathic Medicine, Tallahassee, USA; 2 Research and Academic Affairs, Dow University of Health Sciences, Karachi, PAK; 3 Research and Academic Affairs, Larkin Community Hospital, Miami, USA; 4 Research, Larkin Community Hospital, Miami, USA; 5 Medicine, CES University, Medellin, COL; 6 Faculty of Medicine, Ibn Sina University, Khartoum, SDN; 7 Medicine, Juan N. Corpas University Foundation, Bogotá, COL; 8 Neurology/Osteopathic Neuromuscular Medicine, Larkin Community Hospital, Miami, USA; 9 Interventional Pain, Larkin Community Hospital, Miami, USA; 10 Pain Management, Larkin Community Hospital, Miami, USA

**Keywords:** continuous radiofrequency ablation, osteopathic manipulative medicine, chronic and acute pain management, trigeminal neuralgia, radiofrequency ablation (rfa)

## Abstract

Trigeminal neuralgia (TN) is a chronic pain condition that affects the trigeminal nerve, the largest of the cranial nerves. It is characterized by severe, sudden, and recurrent facial pain, often triggered by light touch or a breeze. Treatment options for TN include medication, nerve blocks, and surgery, but radiofrequency ablation (RFA) has emerged as a promising alternative. RFA is a minimally invasive procedure that uses heat energy to destroy the small portion of the trigeminal nerve responsible for the pain. The procedure is performed under local anesthesia and can be done as an outpatient procedure. RFA has been shown to provide long-term pain relief for TN patients with a low complication rate. However, RFA is not suitable for all TN patients and may not be effective for those with multiple pain sites. Despite these limitations, RFA is a valuable option for TN patients who are not responding to other treatments. Furthermore, RFA is a good alternative for a patient unsuitable for surgery. Further research is needed to fully understand the long-term effectiveness of RFA and identify the best candidates for the procedure.

## Introduction and background

Trigeminal neuralgia (TN) is defined as a chronic condition characterized by the presence of unilateral neuropathic facial pain that involves the fifth cranial nerve and might result in physical and/or psychological disability [1,2]. The prevalence of the disease varies between 0.03% and 0.3% of the population, with an annual incidence of approximately 12 cases per 100,000 individuals [1,3]. While it can occur at any age or gender, a higher incidence has been found in females, with an average onset age of 50 [4,5]. In most cases, the etiology of TN is either idiopathic or caused by external vascular compression of the trigeminal nerve, which results in the deterioration of the myelin layer that protects the nerve. However, any condition that leads to nerve injury can trigger the development of TN (i.e., multiple sclerosis, neural tumors, facial trauma or surgery, and systemic diseases) [1,2].

TN is typically characterized by the presence of sudden, sporadic, intense burning or stabbing-like unilateral facial pain in the distribution of one or more branches of the affected trigeminal nerve [2]. The painful episodes usually last a few seconds to minutes and can occur spontaneously or in response to a non-painful stimulus (e.g., when performing activities involving facial movements) [1,6]. Additionally, the severity of the attacks tends to worsen over time, resulting in fewer pain-free intervals and increased difficulty in symptomatic control [2,4]. The diagnosis of TN is primarily determined by the patient’s medical history and clinical examination findings [4]. However, correctly identifying this condition can often be challenging as the symptoms can be similar to many other conditions that cause facial pain, such as post-herpetic neuralgia, cluster headache, and temporomandibular joint disorder [1,5]. Accordingly, various neurological tests are frequently required to rule out differential diagnoses [5].

The treatment of TN pain can often be achieved through the use of medications such as anti-epileptic drugs or tricyclic antidepressants [4,6]. Nevertheless, conservative treatment might fail to effectively manage the symptoms as the disease progresses [7]. Consequently, there are several interventional approaches for the management of TN, including neurectomy, balloon compression, microvascular decompression, stereotactic radiosurgery, and radiofrequency ablation (RFA) [2,4]. RFA is a modality that applies radio waves directly on the nerve to interrupt the transmission of nociceptive signals [5,8]. It has proven to be an effective treatment strategy for TN due to its excellent therapeutic success rates, minimally invasive nature, and safety profile [1,5,7,8]. RFA can be classified into two subtypes, namely, continuous radiofrequency (CRF) and pulsed radiofrequency (PRF). While CRF is performed by using a steady application of high temperatures to achieve thermocoagulation and necrosis of the targeted tissue, PRF delivers radio waves to the nerve in a pulsed sequence and at lower temperatures [8]. Despite its demonstrated efficacy, RFA can be associated with various risks and complications depending on patient characteristics, TN etiology, and other influencing factors [7,8]. The purpose of this article is to conduct a comprehensive overview of the current literature regarding the utility and indications of RFA for the long-term treatment of intractable TN in the adult population to better understand the role of this minimally invasive modality as a therapeutic alternative for such a complex condition.

## Review

Methods

A series of databases were searched for relevant articles published between January 2019 and December 2022. A review of recent literature indexed in PubMed, Medline, the National Library of Medicine, and Google Scholar was conducted. Our review included 27 retrospective and prospective studies investigating the role of RFA as a treatment strategy for TN. Inclusion criteria included patients >18 years old, with evidence of TN, and receiving RFA therapy. Exclusion criteria included other forms of non-trigeminal facial pain (e.g., migraine, cluster headache, herpes zoster infection, and temporomandibular joint dysfunction). Prospective trials were favored over retrospective studies, and large-scale cohorts were favored over smaller case series.

Discussion

According to the International Headache Society, trigeminal neuralgia (TN) is “a disorder characterized by recurrent unilateral brief electric shock-like pain, abrupt in onset and termination, limited to the distribution of one or more than one division of the trigeminal nerve and triggered by innocuous stimuli” [9]. Although the pathophysiology of TN is not fully understood, the evidence suggests that the pressure on the trigeminal root at the entry zone in pons causes demyelination and leads to an ectopic action potential, which causes neuralgia. TN can be idiopathic or secondary to other disease states, which include multiple sclerosis, cerebellopontine angle tumor, and aberrant vascular loop causing demyelination [10]. In most cases, symptoms begin after the age of 40, with the incidence continuously increasing with age. However, few cases have been reported in the pediatric population. Furthermore, there are no geographical or racial differences in disease incidence [9].

TN is classified into two types, namely, (1) classical TN, purely paroxysmal, and (2) classical TN with concomitant persistent facial pain. Pain is described as superficial, sudden, severe, sharp, lancinating, and shock-like, associated with tic-like cramps of the facial muscle. Triggered by non-noxious stimuli, pain attacks occur several times daily and may worsen over time. Reportedly, the right side of the face is more affected than the left side and has a higher incidence of involvement of the V2 division of the trigeminal nerve than V3 [9,10].

Effective TN treatment started 40 years ago; before that, the most effective treatment was the destruction of the affected nerve branch by the injection of caustic substances or the resection of the sensory trigeminal root behind the Gasserian ganglion [10]. Current treatment therapies include medical management and surgical management. The mainstay for medical management is anticonvulsants, especially carbamazepine or oxcarbazepine. Surgical management is the second line if medical management fails. Failure of medical management is usually due to unacceptable side effects or persistent pain. Types of surgical therapies include microvascular decompression, balloon compression, and radiotherapy [1].

The most invasive surgical approach in treating TN is microvascular decompression, a vascular compression on the trigeminal root as the cause of TN because it removes the suspected nerve compression by the artery or vein [11]. Another type of surgical therapy for TN is balloon decompression. Balloon decompression is a non-invasive procedure wherein a small balloon is passed through a 14-gauge needle to be inflated at the Gasserian ganglion. This mechanism causes a contained injury at the ganglion level that prevents the transmission of pain signals [10]. Another non-invasive approach is RFA, which is the focus of this review [11].

RFA is the most effective minimally invasive technique for treating TN. It has been recognized for its safety, efficacy, success rate, and patient satisfaction compared to balloon and microvascular decompression [10,11]. Therefore, it has become one of the mainstream approaches to treating TN refractory to medications [12]. In addition, RFA at Gasserian ganglion-level intervention may lead to immediate pain relief [13]. A systematic review of the literature, published in 2016, showed that RFA had a success rate of 85-90% in the treatment of TN and a recurrence rate of 5-10%. A meta-analysis of RFA for TN published in 2017, including 13 studies and 1,146 patients, showed that RFA had a success rate of 89.2% and a recurrence rate of 7.9% [9]. In addition to the studies previously mentioned, several other studies have also reported on the effectiveness of RFA for TN. A study published in the Journal of Neurosurgery in 2015, which included 104 patients, reported a success rate of 94% after a mean follow-up of 16.8 months. Another study published in the Journal of Neurosurgery in 2019, which included 100 patients, reported a success rate of 98% after a mean follow-up of 12 months [1]. Moreover, RFA has been found to be cost-effective. A study published in the Journal of Craniofacial Surgery in 2020 reported that RFA was cost-effective compared with microvascular decompression for treating TN [12,13].

RFA may be applied in two ways at the Gasserian ganglion level, namely, thermal radiofrequency or CRF and PRF. The main difference between the two is that thermal RF causes structural damage, whereas PRF does not cause structural damage but can cause microscopic damage to the microfilaments of C-fibers [10]. Both interventions are usually performed under fluoroscopic guidance using a C-arm machine. Most studies used lidocaine subcutaneously for injection analgesia. Aspiration is done before inserting the probe to ensure that there is no cerebrospinal fluid leak or blood vessel injury as the foramen ovale is penetrated. An RF cannula (10 cm, 20-23 gauge with 0.5 cm active tip) was used in patients undergoing RF. The process starts with identifying the coronoid process in the sub-occipitomental view of the C-arm. Once the coronoid process is found, the C-arm is gradually rotated in an ipsilateral oblique position to view the foramen ovale. The depth of the needle is assessed by a lateral C-arm image of the skull with the petroclival junction. If the third division is targeted, the cannula tip is kept 5 mm proximal to the proximal carpal joint (PCJ). If the second division is targeted, the cannula tip is maintained at the PCJ. After placing the cannula in an appropriate position, sensory and motor stimulations are usually done to confirm the placement of the probe. Based on the patient’s response, concordant sensory stimulation is achieved at 0.1-0.5 V/ 50-100 Hz. In all studies, the motor response was checked by testing the corneal reflex after applying 0.1-1.5 V/ 2 Hz stimulation to avoid injuring the motor branch of V3 [10,14].

Newer studies have tried to implement CT guidance instead of fluoroscopy to increase the precision of the procedure. CT guidance was used in these clinical trials as a part of a new approach focusing on targeting the peripheral trigeminal branches instead of the Gasserian ganglion itself [15-18]. However, only a few recent studies compared CT guidance to fluoroscopy. In a systematic review published by Wu et al., a meta-analysis of only two studies concluded that three-dimensional CT guidance and fluoroscopy do not significantly differ in the cure rate.

As the probe temperature during intervention is set between 60°C and 80°C, CRF by high-frequency alternating currents can lead to coagulative tissue necrosis, neuronal injury, and a higher risk of complications. On the other hand, PRF uses short high-voltage bursts, followed by a silent phase, allowing heat elimination. Reduced exposure to heat may decrease the complications, but it will also restrict the effectiveness of therapy. Some studies mentioned that combined CRF and PRF show better results [12,15]. Regarding the duration of the RF application, the data are inconsistent in the amount of time used, as it ranges between 90 seconds to 180 seconds, either in stepwise time intervals or equal time intervals [10,15]. However, Lin et al. have proposed a technique to prevent injuring unnecessary Gasserian ganglion branches; they used stepwise heating from 55°C to 75°C in 5°C increments for 30 seconds each. In this clinical trial, no side effects were associated with injuring unintended trigeminal branches, but only 21 out of 107 patients experienced hematoma [15].

RF temperature is an additional factor that affects the effect of RFA. However, no current standard exists for understanding the accurate CRF temperature required for TN. According to the systematic review, few studies reported that 68°C was the optimal RF temperature for treating the maxillary (V2) and mandibular (V3) division of idiopathic TN and bilateral idiopathic TN. Others mentioned 70°C was the optimal temperature. Patient satisfaction was improved when the temperature range was 68‑70°C, and efficiency was improved at a temperature range of 66-80°C. In other studies, using temperatures of 60-65°C for V1, 72°C for V2, and 75°C for V2/V3 TN resulted in significant patient satisfaction. For PRF, a temperature range of 45-50°C has been recommended for elderly patients. The percentages of patients with initial pain relief were 77.8% to 100%. Pain recurrence was observed in 8-40 months on average [19].

In another study, Wassim et al. treated patients with three lesions at 70° for 60 seconds each for up to three months and reported a 67% rate of recurrence-free interval after the first three months. This rate significantly increased over the years. After one year, 85.3% of patients were symptom-free, 74.6% were symptom-free at three years, and 68.0% at five years, translating to approximately 32% recurrence of symptoms at the end of five years [20]. One study has shown that combination therapy is better and more satisfactory than CRF or PRF alone. The recurrence rate after the procedure is high in lower temperature regimen treatments. The quality of life improvement was observed in all RFA techniques. The resolution of symptoms one year after the procedure ranges between 60% and 95.1% in different studies. At two-year follow-up, patients were more likely to be symptom-free, ranging between 83.3% and 92.3% [21,22].

Despite its effectiveness, RF is not free from complications, including hypoesthesia, masticatory muscle weakness, visual disturbance, keratitis, dysacusis, temporal muscle atrophy, and facial hematoma [20]. In all the studies reviewed, the most presumed side effect was fascial hypoesthesia or numbness, which is expected based on the thermal cauterizing mechanism of RF on the sensory branches of the trigeminal nerve. In the study by Wang et al., the ordered logistic regression model concluded that more severe fascial numbness is associated with previous RF procedure in the affected side (odds ratio (OR) = 2.33, 95% confidence interval (CI) = 1.21-4.48, p = 0.011) and atypical idiopathic TN incidence (OR = 0.36, 95% CI = 0.18-0.71, p = 0.004) [23].

On the other hand, more severe complications such as masticatory muscle weakness, facial hematoma, and corneal reflex weakness are not uncommon. Gunduz et al. have reported that out of 209 patients treated with RF, 19 patients experienced intrabuccal hematoma (9.1%), five patients had masseter muscle weakness (2.39%), and seven patients had either corneal hypoesthesia or weakened corneal reflex (3.35%) [24]. In older literature, more debilitating complications have been documented, including intracranial hemorrhage, intracranial infection, carotid-cavernous fistula, corneal ulcerations, transient cranial nerve III and IV dysfunction, and deafness [25,26]. However, the incidence of these complications has significantly decreased due to the advancements in the needles, imaging, and surgical techniques used to perform the Gasserian ganglion thermocoagulation [25].

Our review concluded that multiple factors might affect the severity of RF complications. For example, Wu et al. focused on the effect of procedure technique on these complications. On comparing PRF vs. CRF, they found that CRF was associated with a higher rate of complications (OR = 0.04; 95% CI = 0.01, 0.23, p = 0.0002) [22]. In another sub-analysis, they also found that CCRF is even safer than CRF. Regarding the temperature used in the procedure, Wu et al. suggested that lower temperatures (68°C-70°C) in CRF are associated with fewer complications when compared with higher temperatures (71°C-75°C) (OR = 0.04; 95% CI = 0.02, 0.09; Z-value 8.17, p < 0.00001) [22]. Despite these promising results, the current RFA practice cannot use the available data to have a specific technique dominant clinically because the existing studies are based on a limited number of cases.

Many of our research studies introduced different approaches to mitigate the potential complications. Huang et al., Zeng et al., and Ran et al. studied the effects of applying the thermal waves extracranially at the affected trigeminal branches (V2, V3) instead of Gasserian ganglion to decrease the risk of intracranial infection or hemorrhage associated with penetrating the cranium [16-18]. Zeng et al. compared PRF via the foramen rotundum (FR) and foramen ovale (FO) vs. Gasserian ganglion radiofrequency (GRF) [17]. They concluded that there was no significant difference in the two-year postoperative effective rate and a great difference in the incidence of numbness between the V2 branch of the PRF group and the GRF group (45 vs. 21, unpaired t-test, p < 0.001) [17]. In addition, Ran et al. studied FR radiofrequency approach to treating V2 idiopathic TN in 87 patients. Post-procedure analysis showed that all patients experienced mild-to-moderate hypoesthesia, and 10 (11.5%) patients experienced transient facial hematoma that resolved after several days [18]. However, none of the patients had masticatory muscle weakness, visual disturbance, meningitis, or corneal areflexia [18]. This extensive safety profile review shows that RFA is proven to be a clinically safe procedure, given that most side effects are transient and non-debilitating. Many variations in the surgical approach have the potential to decrease the side effects. However, more extensive studies and randomized controlled trials are needed to change the current RFA practice.

Although RFA is a minor procedure, some contraindications should be carefully examined. Anesthesia was a determinant factor in a few studies, as patients were excluded if they could not medically tolerate the anesthesia. However, most studies used deep sedation only. Many studies did not include patients to avoid the risk of bleeding and hematoma. Especially in GRF procedures, patients are at a greater risk of sepsis and intracranial infections if they have local facial infections. The most common contraindication to this procedure is a mental or behavioral disease in which patients cannot express subjective feelings because the central part of the procedure is the placement of the needles, which is confirmed by nerve stimulation confirmed by the patient [27].

Although RFA has shown to be an effective treatment for TN, it does have certain limitations. One of the main limitations of RFA is that it is not a cure for TN or other types of chronic pain. RFA can provide significant pain relief but does not address the underlying cause and is not a permanent solution. Sometimes, the pain may return after the procedure, requiring repeat treatments or alternative therapies [18]. Another limitation of RFA is that it is not suitable for everyone. RFA is typically only recommended for patients who have not responded to other forms of treatment, such as medication or surgery.

Additionally, RFA is not recommended for patients with certain medical conditions, such as bleeding disorders or certain tumors [19]. RFA also has potential complications and side effects, such as bleeding, infection, and nerve damage. These risks are generally low but should be considered when evaluating RFA as a treatment option. Moreover, RFA can be expensive and may not be covered by insurance, which can also be a limitation for some patients [15,27]. Overall, RFA is a highly effective treatment option for many types of chronic pain, including TN, but it is not without limitations. It is essential for patients to be aware of these limitations and to discuss them with their healthcare provider before undergoing the procedure.

## Conclusions

RFA is a promising treatment option for patients with TN, providing significant pain relief and improved quality of life. Further studies are needed to compare the long-term outcomes of RFA with other treatments and determine the optimal patient selection criteria. However, it is a safe and effective alternative to other surgical procedures and should be considered a treatment option for patients with TN.
